# Pre-discharge factors associated with early readmission to psychiatric inpatient services within 90 days

**DOI:** 10.1192/bjo.2021.800

**Published:** 2021-06-18

**Authors:** Jessica Wright, Rhianne Thomas

**Affiliations:** Barnet, Enfield and Haringey NHS Mental Health Trust

## Abstract

**Aims:**

The aim of this study was to identify pre-discharge risk factors associated with early inpatient readmission in general adult service users, with a particular focus on modifiable factors. We hypothesised that stability prior to discharge would reduce readmission to inpatient services within 90 days.

**Background:**

Early readmission to inpatient psychiatric services is a poor outcome for service users, staff and the healthcare system. A variety of clinical, demographic and system factors, mostly non-modifiable, have been investigated previously. The identification of pre-discharge and particularly modifiable factors associated with readmission would give an opportunity for intervention and changes in policy.

**Method:**

272 medical records of all admissions within an 8 month period to a NHS inner city psychiatric inpatient service were reviewed to identify factors associated with readmission within 90 days of discharge. The data were analysed by simple comparison, calculation of odds ratios and logistic regression.

**Result:**

26% of service users were readmitted to the mental health trust within 90 days of discharge. Incidents (OR = 3.86; 95% CI 1.39–10.75) and psychotropic medication change in the week before discharge (OR = 2.94; 95% CI 1.43–6.03) were significantly associated with readmission, as were the number of previous admissions, and comorbid substance misuse. Successful overnight leave was found to be significantly protective against readmission (OR = 0.29; 95% CI 0.11–0.72).

**Conclusion:**

The ability to predict those at high risk for readmission means they can be targeted for interventions and it can also help develop best practice around inpatient care and the discharge process. The novel findings in this study of pre-discharge modifiable risk factors such as stability and successful overnight leave could have significant implications in discharge planning policy.

